# Melanocortin 4 receptor signaling in Sim1 neurons permits sexual receptivity in female mice

**DOI:** 10.3389/fendo.2023.983670

**Published:** 2023-03-24

**Authors:** Erin A. Semple, Mitchell T. Harberson, Baijie Xu, Rebecca Rashleigh, Tori L. Cartwright, Jessica J. Braun, Amy C. Custer, Chen Liu, Jennifer W. Hill

**Affiliations:** ^1^ Department of Physiology and Pharmacology, University of Toledo College of Medicine, Toledo, OH, United States; ^2^ Center for Hypothalamic Research, University of Texas Southwestern, Dallas, TX, United States; ^3^ Center for Diabetes and Endocrine Research, University of Toledo, Toledo, OH, United States

**Keywords:** oxytocin, sim1, lordosis, melanocortin, obesity, solicitation, sexual behavior, MC4R

## Abstract

**Introduction:**

Female sexual dysfunction affects approximately 40% of women in the United States, yet few therapeutic options exist for these patients. The melanocortin system is a new treatment target for hypoactive sexual desire disorder (HSDD), but the neuronal pathways involved are unclear.

**Methods:**

In this study, the sexual behavior of female MC4R knockout mice lacking melanocortin 4 receptors (MC4Rs) was examined. The mice were then bred to express MC4Rs exclusively on Sim1 neurons (tbMC4RSim1 mice) or on oxytocin neurons (tbMC4ROxt mice) to examine the effect on sexual responsiveness.

**Results:**

MC4R knockout mice were found to approach males less and have reduced receptivity to copulation, as indicated by a low lordosis quotient. These changes were independent of body weight. Lordosis behavior was normalized in tbMC4R^Sim1^ mice and improved in tbMC4R^Oxt^ mice. In contrast, approach behavior was unchanged in tbMC4R^Sim1^ mice but greatly increased in tbMC4R^Oxt^ animals. The changes were independent of melanocortin-driven metabolic effects.

**Discussion:**

These results implicate MC4R signaling in Oxt neurons in appetitive behaviors and MC4R signaling in Sim1 neurons in female sexual receptivity, while suggesting melanocortin-driven sexual function does not rely on metabolic neural circuits.

## Introduction

Although definitions and assessment tools for the female sexual response cycle are not yet standardized (1–8), available studies have found that approximately 40% of women in the United States report a sexual problem independent of fertility (9–13) and 22% experience distress as a result (9). Few effective options exist for treating sexual dysfunction in women (14–19). One central target that has received attention for the treatment of female sexual dysfunction is the melanocortin system (17). This system consists of three types of neurons: neurons that secrete products of pro-opiomelanocortin (POMC) such as α-melanocyte stimulating hormone (α-MSH), downstream neurons with receptors for those POMC products such as the melanocortin 4 receptor (MC4R), and counter-regulatory neurons that secrete an inverse agonist of the MC4R called agouti-related peptide. α-MSH stimulates lordosis behavior in female rats after administration into the third ventricle, median eminence, medial preoptic area, or ventromedial hypothalamus (20–25). MC3Rs and MC4Rs, found predominantly in the brain (26, 27), facilitate solicitations and lordosis in rodent models (23, 28). In women, bremelanotide—an exogenous melanocortin 3/4 receptor agonist—increases both arousal and sexual satisfaction (29, 30). Intranasal bremelanotide causes adverse side effects such as increased blood pressure, which halted clinical trials (31–33). However, subcutaneous bremelanotide showed sufficient safety and efficacy (30, 34–36) for FDA approval for the treatment of HSDD in patients without uncontrolled hypertension or cardiovascular disease in June of 2019.

The development of bremelanotide is an important step in the treatment of female sexual dysfunction, but the neural pathways underlying its effects on sexual function are unclear. As evidenced by the effects of bremelanotide on blood pressure, centrally acting drugs can cause serious side effects when the neural pathways that they target play additional roles in other systemic functions. To this end, it is imperative that downstream circuitry controlled by melanocortins be explored. We have therefore evaluated the role of the MC4R in the sexual behavior of female mice lacking the receptor throughout the brain.

Expression of the MC4R partially overlaps with that of the transcription factor single-minded homolog 1 (Sim1) in areas such as the paraventricular nucleus of the hypothalamus (PVH), supraoptic nucleus (SON), anterior hypothalamic nucleus, and medial amygdala (MeA) (37). These areas are critical targets of POMC neuronal projections. We hypothesized that Sim1-expressing neurons might mediate the MC4R-dependent effects of melanocortins on female sexual function. To test this possibility functionally, we produced tbMC4R^Sim1^ mice that only express MC4Rs in Sim1 neurons. In addition, Sim1-expressing neurons in regions such as the PVH and SON have been shown to include oxytocin neurons (38, 39). Oxytocin promotes sexual behavior and contributes to sexual desire and reward (40–45). In humans, plasma oxytocin levels increase during sexual arousal and orgasm in both women and men (46). Therefore, we also examined mice expressing MC4R only in oxytocin neurons to determine whether this population of neurons is sufficient for melanocortin-mediated sexual behavior.

## Materials and methods

### Animal production and care

MC4R knockout mice on a C57BL/6 background, originally produced by the Lowell lab, were obtained from The Jackson Laboratory (loxTB Mc4r, 006414). These mice contain a floxed transcription blocker in the MC4R gene promoter that prevents MC4R transcription. Tissue-specific cre expression results in the removal of the transcription blocker, and thus the expression of MC4R. Littermate wild-type (WT) mice with normal MC4R expression were generated from crosses of heterozygous loxTB-MC4R parental mice. Homozygous loxTB-MC4R (MC4RKO) offspring were compared to these WT littermates unless otherwise specified.

To target cre expression to Sim1-expressing neurons (47–49), we used Sim1-cre mice (The Jackson Laboratory, 006395). We bred mice to be homozygous for the floxed MC4R and hemizygous for the Sim1-cre genes, resulting in the expression of MC4R only on Sim1-expressing neurons (tbMC4R^Sim1^). To assist with visualization of Sim1-expressing neurons, these mice were also bred with mice expressing a cre-dependent tdTomato reporter (The Jackson Laboratory, 007909). In a parallel set of experiments, an oxytocin-cre mouse line was used to generate mice that express MC4R only on oxytocin neurons (tbMC4R^Oxt^). Genotyping was confirmed by automated qPCR by Transnetyx, Inc. The specificity of cre expression in Sim1-cre and oxytocin-cre mice has been demonstrated in the literature, and tbMC4R^Sim1^ is a well-established mouse model (47, 50–52).

All experimental protocols were approved by the University of Toledo’s Institutional Animal Care and Use Committee (IACUC), and the mice used in this study were kept in accordance with IACUC guidelines. The sex of mice was determined at weaning by anogenital distance. The mice were housed in the facilities of the Department of Laboratory Animal Resources, where they were given *ad libidum* access to food and water on a 12:12 light-dark cycle. Except for mice that received high fat diet (described below), mice ate standard rodent chow (2016 Teklad Global 16% Protein Rodent Diet, 12% fat by calories; Harlan Laboratories, Indianapolis, Indiana).

### Sexual behavior studies

To allow induction of estrus with hormonal treatment, female mice were ovariectomized between five to six weeks of age and given a week to recover. Previous studies in mice have found that inexperienced females are much less likely to exhibit lordosis (53, 54). Thus, beginning at week seven to eight, these female mice were paired with experienced C57BL/6 male mice on four separate occasions to gain sexual experience. All males used in pairings and experiments had at least four sexual experiences and carried no genetic modifications. Females were hormonally primed to induce behavioral estrus using 100µL of subcutaneous β-estradiol-3-benzoate in sesame oil (200µg/mL) 48 hours prior to pairing followed by 125 µL of intraperitoneal progesterone (4mg/mL) seven hours prior to pairing (55). Females were paired with experienced male mice from 8pm-9am, during their normal period of activity.

Experimental mice were two months of age at the time of the experimental pairing. The pairing was filmed (DVR Swann 4500 and T850 Day and Night Security Camera security system) between 8pm and 12am and later analyzed for behaviors prior to the first ejaculation, if any. Each pair was placed in a separate cage and in a dark room during experimentation. Female sexual behavior includes receptive behaviors, such as lordosis, as well as expressions of sexual motivation through appetitive or proceptive behaviors designed to solicit the attention of the male. Rejection behaviors were also recorded including bucking, biting, and pushing back against the male. Preliminary studies established that mouse solicitation and lordosis behaviors differ from those of rats and require modified criteria for evaluation, as found by others (56). Approaches, consisting of nose-to-nose and anogenital sniffs of the male, were considered to be expressions of social and/or sexual interest by the female. The following criteria were used to identify lordosis: all four paws of the female firmly planted on the ground with the front half of the mouse pushed up off the ground. The lordosis quotient, the frequency of lordosis behavior during male mounting, was calculated as the number of lordosis behaviors divided by the number of mounts (x100). Videos were blinded and one observer scored all videos to reduce inter-observer variability.

### Metabolic testing

**Figure 1:**
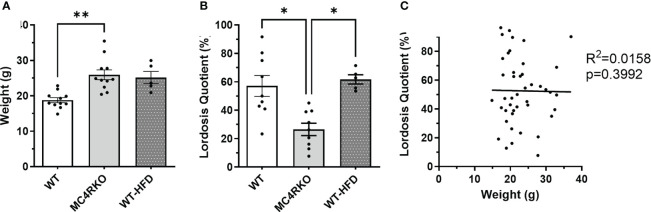
Mice fed HFD had normal sexual function at two months of age. **(A)** Weight of WT (n=11), MC4RKO (n=11), and WT mice on HFD (WT-HFD) (n=5). Brown-Forsythe and Welch ANOVA with Dunnett’s T3 multiple comparisons test. WT vs MC4RKO (p=0.003) and WT-HFD (p=0.101). **(B)** Lordosis quotient between WT (n=9), WT-HFD (n=5), and MC4RKO mice (n=9). One-way ANOVA with Sidak’s multiple comparison test was used: WT vs MC4RKO (p=0.018); MC4RKO vs WT-HFD (p=0.023). **(C)** All mouse groups were combined (n=44) to run correlation analysis on weight vs lordosis quotient. *p<0.05; **p<0.01.

MC4R null mice exhibit obesity (47, 57), so sexual behavior was examined at two months of age, before obesity became a confounding factor. At this age, all mice weighed below 35g (**Figure 1A**). Glucose tolerance testing (GTT), nuclear magnetic resonance (NMR), and calorimetric cages were used to assess the metabolic profile of these mice. Specifically, the morning after sexual behavior testing, NMR was used to assess body composition (BrukerOptics). Then, at 9-12 weeks old, a GTT was performed. The morning of the GTT, mice were fasted for six hours on alpha-dri bedding. Following the fast, baseline glucose levels were obtained from tail-vein blood samples with an AlphaTRAK 2 (ADW Diabetes) glucometer. A 2g/kg dosage of dextrose was given intraperitoneally, and blood glucose levels were measured at 15, 30, 45, 60, 90, and 120 minutes post-injection. After completion of the sexual behavior studies and other metabolic measurements (10 - 12 weeks old), mice were individually housed in calorimetric cages (OxyMax system, Columbus Instruments, Columbus, OH). Metabolic parameters and food intake were measured over a 3-day period after one day of acclimation. Mice had access to food and water ad libitum. Spontaneous locomotor activity was measured with an optical beam measuring horizontal and vertical movement (XYZ-axis). Oxygen consumption (VO_2_), carbon dioxide production (VCO_2_), and energy expenditure were sampled every 20 min. All calculations were made with OxyMax software, with the modified Weir equation used to determine energy expenditure.

### Anxiety-like behaviors

Two tests of anxiety-like behavior were video recorded for analysis by a single observer. For the elevated plus maze, mice were placed in the center of a standard apparatus and allowed to explore for 10 minutes. Two paws in an arm of the maze were recorded as an entry. For the open field test, animals were placed into the center of a 4x4 grid of squares with four center squares and surrounding outside squares. An entry into a box was counted when at least three paws were placed in it simultaneously.

### Diet-induced obesity

For DIO tests, WT littermates were given a high fat diet (HFD) (OpenSource Diets, 60% fat content) instead of standard chow at five weeks of age. Mice were weighed weekly until their weights were comparable to MC4RKO mice at the time of their sexual behavior testing.

### Serum hormone concentrations

Submandibular blood samples were taken between 10 to 13 weeks of age from ovariectomized mice without sex hormone replacement. Blood was spun down in the centrifuge for 10 minutes at 4°C at 4472 RCF. Serum was collected and stored at -80°C until analysis. Luteinizing hormone (LH) and follicle-stimulating hormone (FSH) were collected for measurement from ovariectomized animals two weeks after behavioral testing (no steroid replacement) by the University of Virginia Ligand Assay Core using the Milliplex Pituitary Magnetic Bead Assay (RPTMAG-86K; Millipore), with a combined intra-assay CV of 5.5% and an inter-assay CV of 11.5%. Samples were run in duplicate. The lower limit of detection for the LH multiplex assay was 0.24 ng/ml. The lower limit of detection for the FSH multiplex assay was 0.48 ng/ml.

### *In situ* hybridization

**Figure 2:**
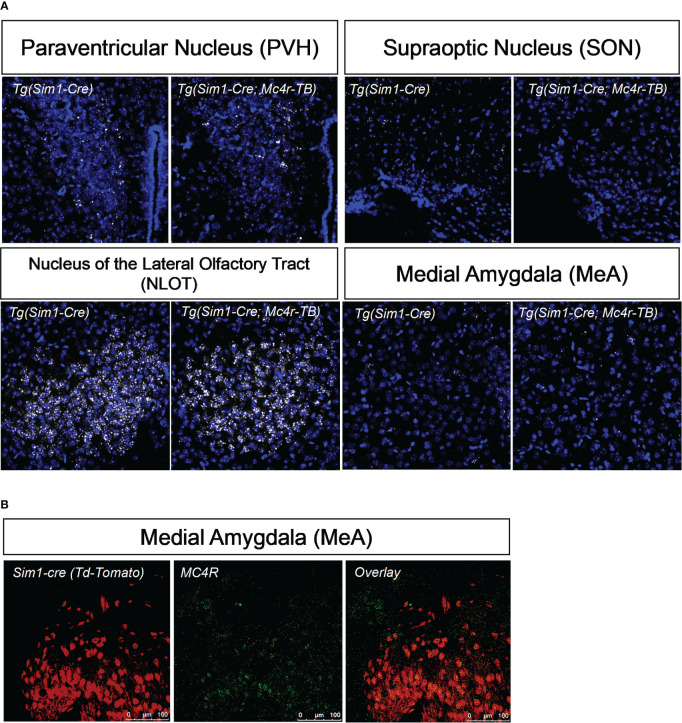
MC4R re-expression and colocalization with Sim1. **(A)** Using *in situ* hybridization, mRNA of MC4R in tbMC4R^Sim1^ mouse brains was confirmed in the paraventricular hypothalamus (PVH), the nucleus of the olfactory tract (NLOT), and the medial amygdala (MeA). MC4R mRNA is labelled white. Nuclei were labelled using DAPI (blue). **(B)** Using immunohistochemistry, co-localization between MC4R and Sim1-cre driven tdTomato expression was confirmed in the MeA of Sim1-cre mice. Sim1 (tdTomato) labelled red; MC4R labelled green.

For the Sim1-cre and tbMC4R^Sim1^ mice in **Figure 2**, mouse brains were fixed in 4% paraformaldehyde overnight and then cut into five series of 25 μm sections with a vibrating-blade microtome (VT1000S, Leica). MC4R mRNA detection was achieved using an MC4R RNAscope probe (ACD, #319181), Opal 690 dye (1:1000), and detection kits (ACD, #323100) from Advanced Cell Diagnostics following the manufacturer’s protocol. All fluorescence images were acquired using a Zeiss LSM880 Airyscan confocal microscope.

**Figure 3:**
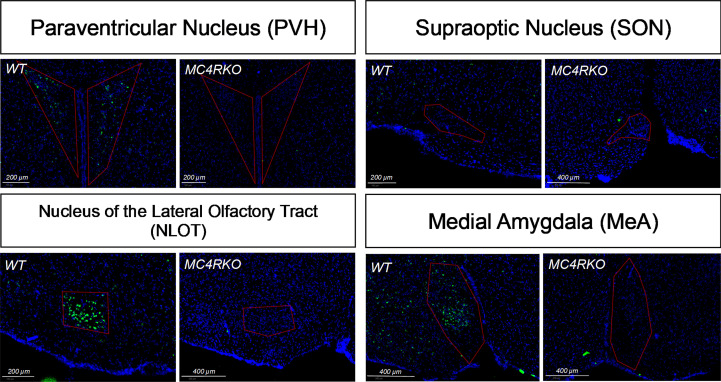
*In situ* hybridization showing lack of MC4R in MC4RKO mice in the paraventricular hypothalamus (PVH), the nucleus of the olfactory tract (NLOT), and the medial amygdala (MeA). MC4R mRNA is labelled green. Nuclei were labelled using DAPI (blue).

**Figure 4:**
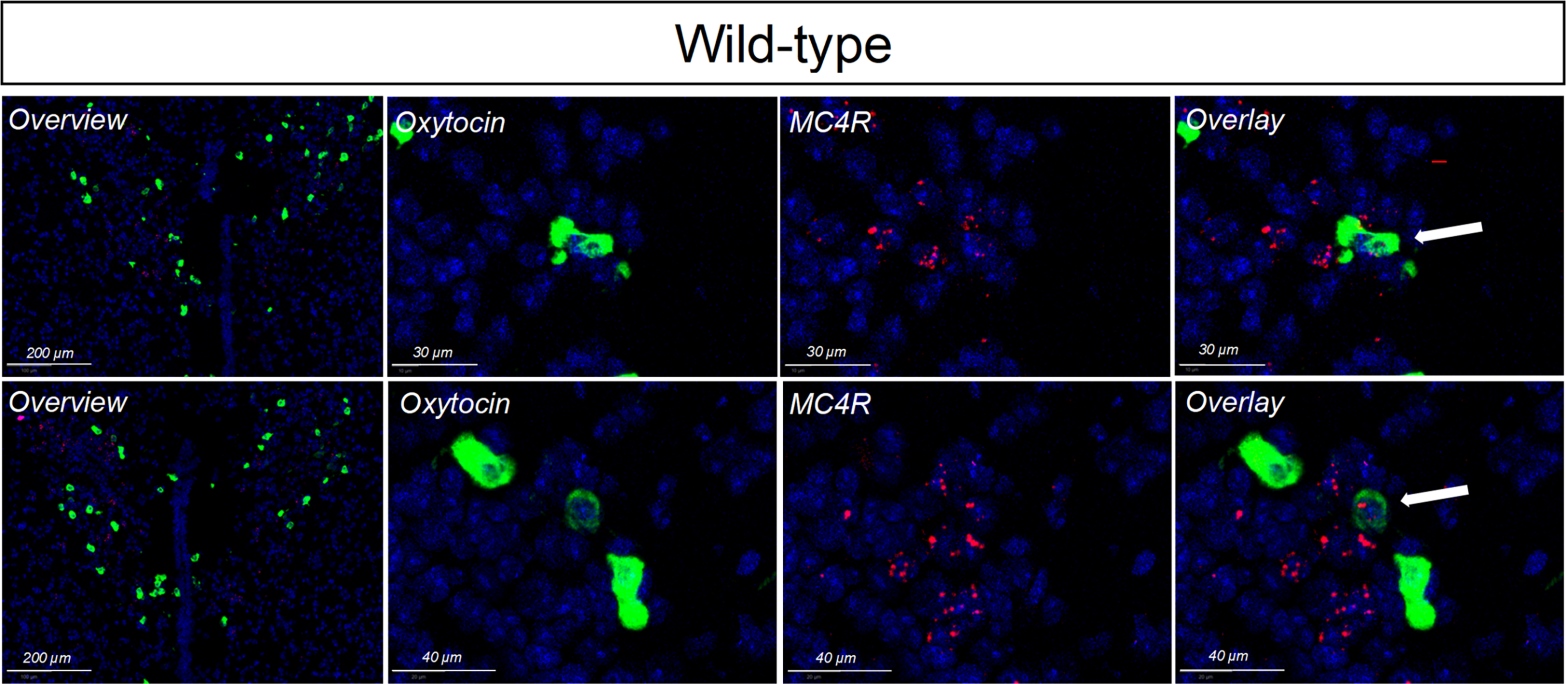
*In situ* hybridization for oxytocin (labeled in red) and MC4R (labeled in green) in the PVH of wildtype mice. Nuclei were labelled using DAPI (blue). Limited, but detectable, overlap was seen.

For the WT and MC4RKO mice in **Figures 3** and **4**, mouse brains were perfused with phosphate buffered saline (PBS) for 2 minutes, frozen on dry ice, cut into 14 um sections using a cryostat (Leica CM3050 S), and adhered to Superfrost Plus Microscope Slides (Fisher Scientific). mRNA was detected using the protocol and reagents in the RNAscope Multiplex Fluorescent V2 Assay from ACD. A custom oxytocin probe (ACD, #1240951-C1) was used with the fluorophore Opal 570 (1:6000), and the MC4R probe (ACD, #319181) was used with the fluorophore Opal 690 (1:500). For **Figure 3**, full brain sections were scanned at 20X using an Olympus VS120 slide scanner microscope. For **Figure 4**, the PVH and SON were imaged using a Leica TCS SP5 Confocal Microscope. Each image was set to the same display settings prior to image capture.

### Immunohistochemistry

Upon euthanasia, mice were perfused with 4% paraformaldehyde prior to brain harvesting. The brains were transitioned to a 10% sucrose, a 20% sucrose, and finally a 30% sucrose solution in PBS, waiting until the tissue sank prior to each transition. The brain was sectioned in 35-40µm slices and then stored in cryoprotectant (20% glycerol and 30% ethylene glycol in PBS) until immunohistochemistry was performed. To examine MC4R localization on Sim1 neurons, brain sections were labeled overnight with rabbit anti-MC4R (Abcam ab24233) and then exposed to a Donkey anti-rabbit IgG Alexa Fluor 488 secondary antibody (Invitrogen, A21206). TdTomato was used as a cre-reporter; its fluorescence was visible *via* confocal (Leica TCS SP5) without the aid of an additional antibody.

### Statistical analysis

Data analysis was performed using Graphpad 9.4.1. Data with only two groups were analyzed using an unpaired t-test. An F test was used to compare the variances between these groups, and when the variances were not equal, the t-test was run with Welch’s correction. Comparisons of more than two groups were made using a one-way ANOVA with Sidak’s multiple comparisons test. Groups were tested for equal variances using Levene’s test. When data sets did not have equal variances, they were analyzed using Brown-Forsythe and Welch ANOVA with Dunnett T3’s multiple comparisons test. All groups were tested for normality using the Kolmogorov-Smirnov test. Groups that were not normally distributed were accepted if the skewness and kurtosis were within +/-3. When a group had kurtosis higher than three, outliers were identified using ROUT (Q = 1%) analysis and removed to reduce the kurtosis of all data sets to below three. Correlation tests were used to determine the relationship between two continuous variables, with results reported as R^2^ values. Data in bar graphs are presented as mean ± SEM (95% confidence interval). Statistical significance was defined as p<0.05. In figure legends, *, p<0.05; **, p<0.01; ***, p<0.001.

## Results

To verify the deletion of MC4R in our female MC4RKO mice, mRNA in coronal brain sections was detected using RNAscope *in situ* hybridization. Compared to WT sections, MC4RKO mice lacked mRNA for MC4R in the PVH, the nucleus of the lateral olfactory tract (NLOT) and the MeA (**Figure 3**). Although MC4R expression in the SON has been reported (37), we found MC4R expression there was too low to be detected in the WT mice (**Figure 3**).

**Figure 5:**
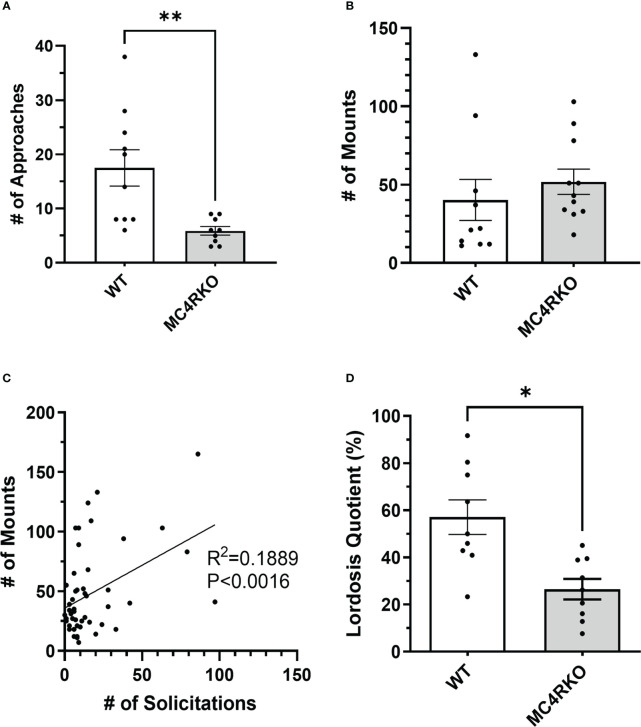
MC4R deletion resulted in fewer approach behaviors and a lower lordosis quotient compared to the WT control. Female approaches towards the male (sniffs or touches) **(A)**, Male mounts **(B)**, and lordosis quotient **(D)** are compared between WT (n=9) and MC4RKO (n=9-11). All mouse groups were combined (WT, Sim1-cre, Oxt-cre, MC4RKO, tbMC4R^Sim1^, tbMC4R^Oxt^; n=50) to run correlation analysis on approaches vs mounts **(C)**. Groups in this figure were statistically analyzed together with groups from **Figure 6**. One-way ANOVA with Sidak’s multiple comparisons test was used for graphs A and C. Brown-Forsythe and Welch ANOVA tests with Dunnett’s T3 multiple comparisons test was used for graph B. Graph A, p=0.008. Graph B, p=0.018 *p<0.05; **p<0.01.

To characterize the sexual behavior of female MC4RKO mice, females were ovariectomized, primed with estradiol and progesterone before all pairings, and given four sexual experiences with sexually experienced, control males before experiments began at 8-10 weeks of age. We quantified approach behaviors that show female interest in the male, rejection behaviors, mounts by the male, and the assumption by the female of a reflexive lordosis posture that indicates receptivity. Female approach behaviors were significantly reduced in MC4RKO mice compared to WT controls (p=0.043; **Figure 5A**). 10% of WT mice showed a rejection behavior such as attempting to bite or hit the male (1 out of 10) vs 45.4% percent of MC4RKO mice (5 out of 11; data not shown). In contrast, the number of times females were mounted by their male partners did not differ, indicating male behavior was consistent between groups regardless of female sexual motivation (**Figure 5B**). When examining data from all animals used in these studies, we found that the number of male mounts positively correlated with the number of female approaches; however, this effect accounted for only 18.9% of the overall variance in male mounting frequency (**Figure 5C**), too small to produce a detectable difference between the WT and MC4RKO groups. Finally, MC4RKO females had a decreased lordosis quotient (p=0.018; **Figure 5D**). This finding indicates that the MC4RKO mice were less sexually receptive than control animals.

**Figure 6:**
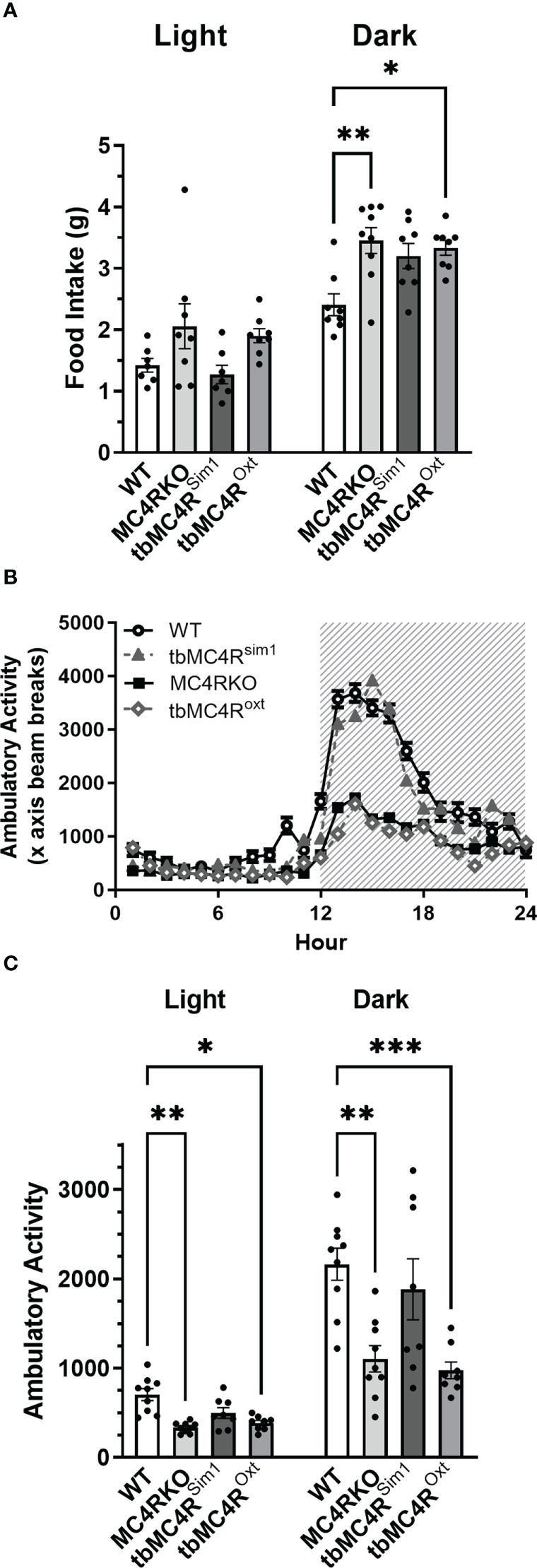
Expression of the MC4R on Sim1 neurons normalizes food intake and locomotion. Food intake in day and night **(A)**, ambulatory activity along the x-axis **(B)**, and area under the curve for diurnal and nocturnal ambulatory activity **(C)** as measured in calorimetric cages for WT (n=7-9), MC4RKO (n=8-9), tbMC4R^Sim1^ (n=7-8), and tbMC4R^Oxt^ mice (n=8). One-way ANOVA with Sidak’s multiple comparisons test was used for graph A. Brown-Forsythe and Welch ANOVA tests with Dunnett’s T3 multiple comparisons test was used for graph C. Graph A night, WT vs MC4RKO (p=0.003) and tbMC4R^Oxt^ (p=0.018). Graph C day, WT vs MC4RKO (p=0.005) and tbMC4R^Oxt^ (p=0.012). Graph C night, WT vs MC4RKO (p=0.004) and tbMC4R^Oxt^ (p=0.0008) *p<0.05; **p<0.01; ***p<0.001.

**Figure 7:**
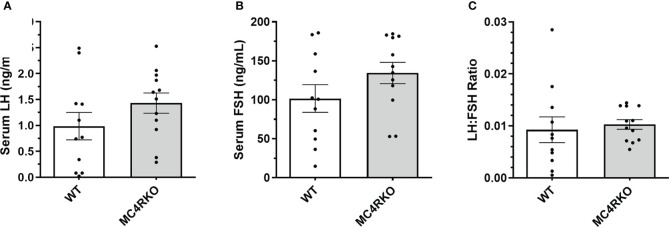
Serum LH and FSH concentrations were not different between groups. LH **(A)** and FSH **(B)** are represented separately as well as in the form of an LH : FSH ratio **(C)** for WT (n=11) and MC4RKO (n=12). Unpaired t-tests were used for graphs A and B. Unpaired Welch’s t-test was used for Graph C.

Gonadotropin-releasing hormone (GnRH) can facilitate lordosis behavior (58). One previous study found that LH concentrations were altered between four and seven months of age in intact MC4R null mice (59). In addition, POMC neurons have been hypothesized to play a role in sex steroid negative feedback (60). Any resulting altered GnRH and LH profile could have implications for sexual receptivity even in gonadectomized animals. We therefore examined these sex hormones in serum to determine whether the genotype affected how the hypothalamic-pituitary axis responded to ovariectomy. No differences were seen in LH and FSH concentrations or the LH/FSH ratio among the groups (**Figures 7A–C**).

**Figure 8:**
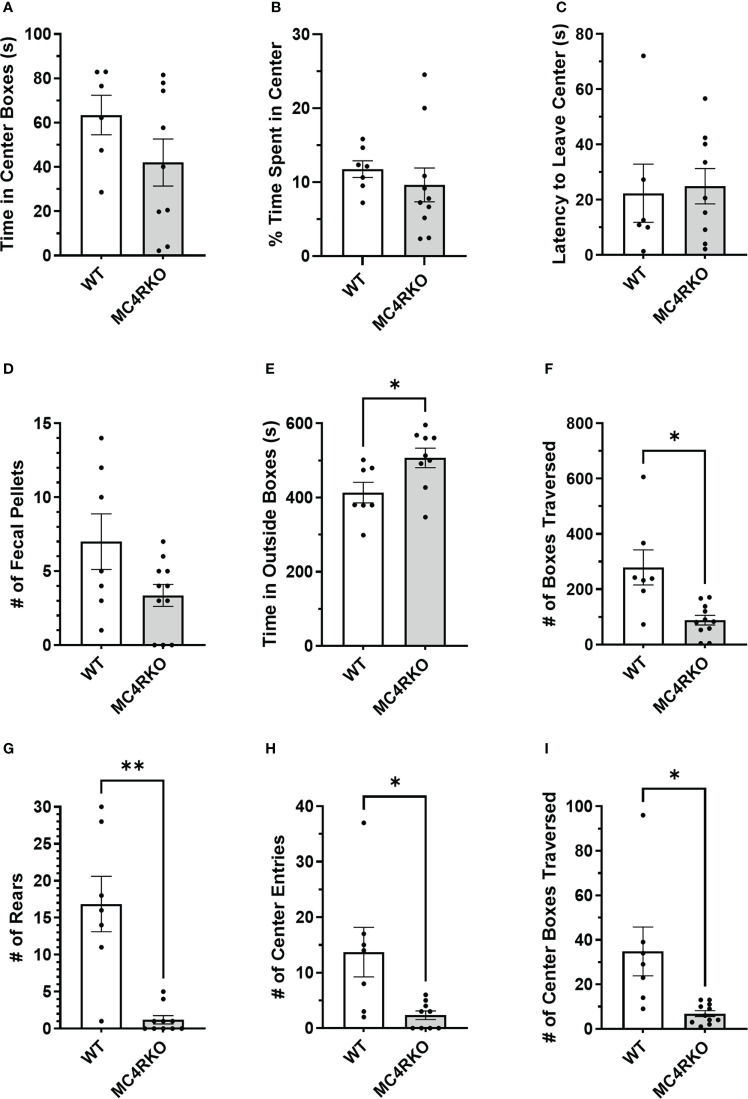
MC4RKO mice exhibit decreased locomotion in an open field test. The time that each animal spent in the center boxes **(A)**, the percent of time spent in the center **(B)**, the latency to leave the center **(C)**, the number of fecal pellets produced during the test **(D)**, time spent in the outside boxes **(E)**, the number of boxes traversed **(F)**, the number of rears **(G)**, the number of entries into the center boxes **(H)**, and the number of center boxes traversed **(I)** is shown. WT=6-7 and MC4RKO=9-11 mice. Unpaired t-tests were used for graphs D-F and K; Unpaired Welch’s t-tests were used for graphs G-J. Graph F (p=0.029); Graph G (p=0.023); Graph H (p=0.006); Graph I (p=0.044); Graph J (p=0.0433). *p<0.05; **p<0.01.

Because sexual function can be negatively affected by anxiety, we conducted an open field test to see if loss of MC4R increased anxiety-like behaviors. In the open field test, WT and MC4RKO mice showed no difference in latency to leave the center (**Figure 8A**). They also did not differ in the absolute time or percentage of time they spent in the center boxes (**Figures 8B, C**). WT and MC4RKO mice also had no difference in the number of fecal pellets produced during the test, another measure of anxiety (**Figure 8D**). However, a small but significant increase was observed in the amount of time MC4RKO mice spent in the outside boxes (p=0.029; **Figure 8E**). MC4RKO mice showed decreased locomotion and activity as indicated by the fewer number of boxes traversed (p=0.023) and number of rears (p=0.0056; **Figures 8F, G**). This finding was accompanied by a decrease in the number of entries into the center and the number of center boxes traversed (**Figures 8H, I**).

**Figure 9:**
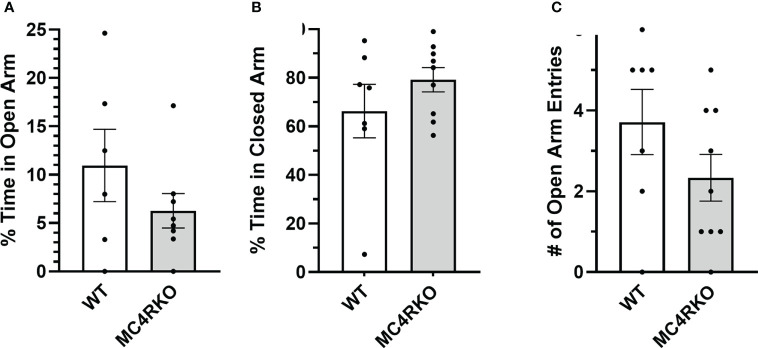
MC4RKO mice show no anxiety in an elevated plus maze. Time spent in the open **(A)** and closed **(B)** arms and entrances into the open arms **(C)** of an elevated plus maze by WT (n=6-7) and MC4RKO mice (n=8-9). No significant differences were found using unpaired t-tests.

The results of the open field test indicated that MC4RKO mice may have higher levels of anxiety or less locomotor activity. We therefore performed a second test for anxiety-related behaviors. In an elevated plus maze, WT and MC4RKO mice spent an equal percentage of time in the open and closed arms and had an equal number of entries into the open arms (**Figures 9A–C**). These results argue against the idea that MC4RKO mice exhibit increased anxiety.

**Figure 10:**
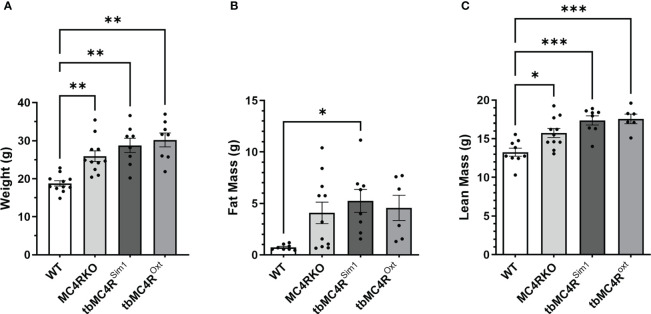
Body composition of mice lacking MC4R. Comparison of the **(A)** body weight, **(B)** fat mass, **(C)** lean mass measured by NMR of cre control (n=8-11), MC4RKO (n=9-11), tbMC4R^Sim1^ (n=8), and tbMC4R^Oxt^ (n=6-9) groups at two months of age. One-way ANOVA with Sidak’s multiple comparisons test was used for graphs C and E. Brown-Forsythe and Welch ANOVA tests with Dunnett’s T3 multiple comparisons test was used for graphs A and B. Graph A, WT vs MC4RKO (p=0.003), tbMC4R^Sim1^ (p=0.005), and tbMC4R^Oxt^ (p=0.002). Graph B, WT vs tbMC4R^Sim1^ (p=0.025). Graph C, WT vs MC4RKO (p=0.019), tbMC4R^Sim1^ (p=0.0002), and tbMC4R^Oxt^ (p=0.0003). Graph E, MC4RKO vs tbMC4R^Sim1^ (p=0.007) *p<0.05; **p<0.01; ***p<0.001.

**Figure 11:**
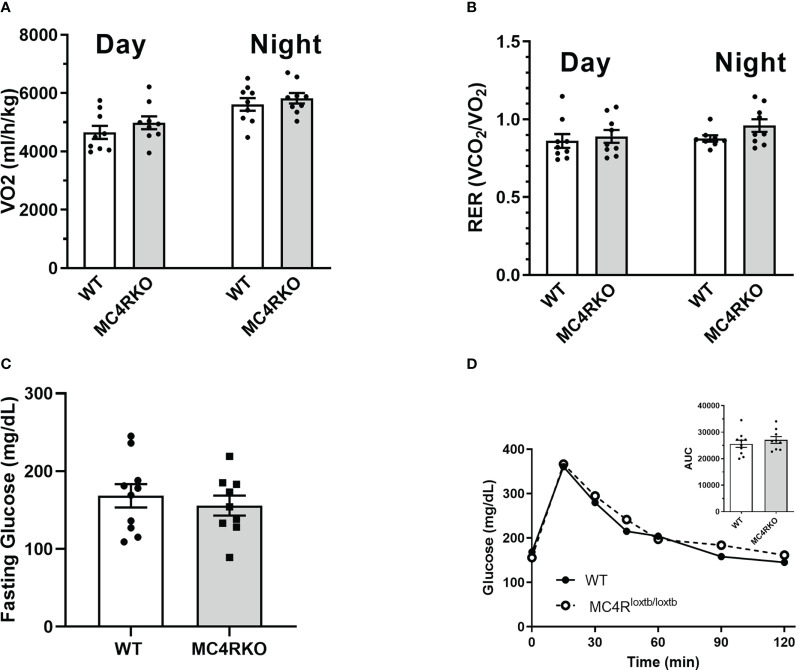
Unchanged metabolic rate and glucose sensitivity in MC4RKO females. Oxygen consumption **(A)** and respiratory exchange ratio **(B)** during the day and night measured from WT (n=7-9) and MC4RKO (n=8-9) mice in calorimetric cages. Glucose levels in mice after a 6 hour fast **(C)** and after glucose administration during a glucose tolerance test **(D)**, with the areas under the curves shown in the inset. Unpaired t-tests were used for all graphs.

MC4RKO mice are well-known to exhibit a hyperphagic phenotype accompanied by energy-conserving adaptive changes, such as reduced locomotion around their cages (61). We therefore acquired a metabolic profile on these animals at 9-12 weeks of age. MC4RKO mice had significantly increased weight gain compared to WT (p=0.041) (47), as well as a significant increase in lean mass (p=0.019), but surprisingly not fat mass, although a trend was evident (p=0.052; **Figures 10A–C**). In addition, MC4RKO mice showed increased food intake in the dark phase (p=0.0033; **Figure 6A**) and substantially decreased locomotor activity in both the day (p=0.0053) and night (0.0042; **Figures 6B, C**), as previously observed in the open field test. We did not detect a difference in oxygen consumption, respiratory exchange ratio, fasted glucose levels, or glucose tolerance (p=0.4478) (**Figures 11A–D**). These findings are consistent with previous work that found no hyperglycemia in female MC4R null mice (62), although another study found hyperglycemia in this model secondary to increased adiposity (63).

While all mice weighed less than 35 grams in this study, we considered the possibility that increased body weight contributed to their sexual phenotype. To examine the effect of adiposity on the sexual receptivity of mice, we generated diet-induced obesity (DIO) mice. WT mice were fed a high fat chow diet starting at five weeks of age and ending at two months of age when their weights were comparable to MC4R null mice (p>0.9999; **Figure 1A**). As in **Figure 5**, females were ovariectomized, primed with estradiol and progesterone before all pairings, and paired with sexually-experienced, control males for four practice matings and the recorded experiment. As previously mentioned, MC4RKO mice had a significantly lower lordosis quotient compared to WT mice. However, DIO mice exhibited a lordosis quotient comparable to WT mice (p>0.9999) and significantly higher than MC4RKO mice (**Figure 1B**; p=0.023). Correlation analysis confirmed that body weight did not correlate with lordotic response in the mice studied (p=0.94; **Figure 1C**). These data show that the sexual behavior deficits of young MC4R null mice cannot be attributed to weight gain.

Single-minded 1 (Sim1) is a transcription factor involved in hypothalamic development. Because Sim1 neurons express MC4R (48, 49), we were interested in whether these neurons are involved in the actions of melanocortins on female sexual behavior. In addition, MC4R activation has been shown to increase hypothalamic oxytocin levels and increase social investigation in male mice; these changes were blocked by an oxytocin receptor antagonist (64). We therefore hypothesized that Sim1-expressing neurons and/or oxytocin-expressing neurons might mediate the MC4R-dependent effects of melanocortins on sexual receptivity of female mice. To test this possibility, we produced tbMC4R^Sim1^ mice that express MC4R only on Sim1 neurons and tbMC4R^oxt^ mice that express MC4R only on oxytocin neurons. Expression of MC4R in tbMC4R^Sim1^ animals was confirmed using *in situ* hybridization. The amount of mRNA for MC4R was similar between Sim1-cre and tbMC4R^Sim1^ in the PVH, SON, NLOT, and MeA (**Figure 2A**). Since MC4R expression in the MeA was light, we verified the presence of MC4R in Sim1 using immunohistochemistry. After crossing the line with a cre-dependent tdTomato reporter line, we found that MC4R co-localized with Sim1-tdTomato positive neurons in the MeA (**Figure 2B**). In tbMC4R^Oxt^ mice, RNAscope experiments demonstrated a small amount of oxytocin and MC4R co-expression that varied depending on the coronal plane examined (**Figures 4A, B**). While oxytocin expression in the SON was robust (data not shown), we were unable to detect MC4R expression in that area (**Figure 3**). Very few oxytocin neurons were seen outside the PVH and SON.

Compared to cre controls, and similar to MC4RKO mice, tbMC4R^Sim1^ mice had significantly increased weight (p=0.0002), fat mass (p=0.0254), and lean mass (p=0.0002; **Figures 10A-C**). These findings contrast with a previous study that found attenuated weight gain in tbMC4R^Sim1^ mice (58), although in that study the mice had not been ovariectomized prior to testing. Likewise, tbMC4R^Oxt^ mice also had significantly increased weight (p<0.0001) and lean mass (p=0.0003) but not fat mass (p=0.011) compared to controls, with no significant difference from MC4RKO animals (**Figures 10A–C**). Thus, the reexpression of MC4Rs in Sim1 or Oxt neurons was unable to alter female body weight at this age. On the other hand, the increased food intake seen in MC4RKO animals was rescued in tbMC4R^Sim1^ but not tbMC4R^Oxt^ mice (p=0.018; **Figure 6A**). The reduced activity of MC4RKO mice was likewise rescued in tbMC4R^Sim1^ but not tbMC4R^Oxt^ mice (Dark p=0.0008; **Figures 6B, C**). These findings largely confirm previously published information on these models (47–49, 65). It is probable that decreased calorie intake and increased expenditure through locomotion would eventually lead to a reduction in body weight compared to MC4RKO mice in tbMC4R^Sim1^ animals.

**Figure 12:**
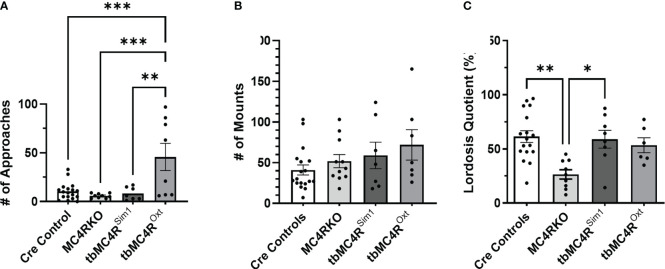
MC4R expression exclusively on Sim1 neurons resulted in a lordosis quotient comparable to the Sim1-cre control. Female approaches to the male **(A)**, male mounts **(B)**, and lordosis quotient **(C)** were compared between combined cre controls (n=17-18), MC4RKO (n=9-11), tbMC4R^Sim1^ (n=7-8), and tbMC4R^Oxt^ mice (n=6-7). One-way ANOVA with Sidak’s multiple comparisons test was used for graphs A and C. Brown-Forsythe and Welch ANOVA tests with Dunnett’s T3 multiple comparisons test was used for graph B. Graph A, tbMC4R^Oxt^ vs Cre Controls (p=0.0003) and MC4RKO (p=0.0003) and tbMC4R^Sim1^ (p=0.0017); Graph C, MC4RKO vs Cre Controls (p=0.0009) and tbMC4R^Sim1^ (p=0.011) and tbMC4R^Oxt^ (p=0.069) *p<0.05; **p<0.01; ***p<0.001.

The sexual behaviors of tbMC4R^Sim1^ mice and tbMC4R^Oxt^ mice were assessed in comparison to mice carrying only the Sim1-cre or the Oxt-cre allele. As these two control groups did not differ significantly on any measure, their data were pooled. As shown in **Figure 12A**, approach behaviors did not differ among the cre controls, MC4RKO, and tbMC4R^Sim1^ mice, although the MC4RKO group showed the lowest mean number. Interestingly, approaches were dramatically elevated in the tbMC4R^Oxt^ group, with 4 out of 7 animals showing more than 50 approaches toward the male during the recording period. Only 11.8% of cre control mice showed a rejection behavior (2 out of 17) compared to the 45.5% percent of MC4RKO mice previously seen. No tbMC4R^Sim1^ females attempted to fight the male (0 of 7), while 25% of tbMC4R^Oxt^ females did so (2 out of 8). Aggressive mice displayed a range of approach frequencies from low to high (data not shown), suggesting social interest shown towards the male did not always translate into sexual interest. Mounting rates were consistent across all groups (**Figure 12B**). tbMC4R^Sim1^ mice displayed a lordosis quotient that was consistent with that of cre controls and significantly higher than MC4RKO mice (**Figure 12C**, p=0.0079). This result indicates that expression of MC4R on Sim1 neurons alone is sufficient to permit normal lordosis behavior. In contrast, the lordosis quotient of tbMC4R^Oxt^ females was intermediate between that of the cre controls and MC4RKO mice, significantly different from neither (**Figure 12C**). Thus, expression of MC4R only on Oxt neurons appears to promote social and/or sexual interest while having a lesser impact on the promotion of lordosis.

## Discussion

### Melanocortins and the female sexual response

The current study investigated the specific neurons involved in the effect of MC4R function on sexual behavior in female mice. To clarify the role of the MC4R, we used a mouse model in which MC4R transcription is blocked globally and assessed the effect on female sexual receptivity at 2 months of age. To the best of our knowledge, this is the first time that the sexual behavior of female MC4R null mice has been tested. We found that compared to littermate controls, female MC4R knockout mice showed less interest in the male and reduced sexual receptivity, as measured by a significantly decreased number of approach behaviors and reduced lordosis quotient respectively. These data add to the growing evidence that POMC neurons play an important role in sexual behavior and fertility. These results are also in accord with the ability of exogenous melanocortins to promote sexual behavior and function through the MC4R in many species including humans.

Potential sexual solicitation behaviors by female mice deserve more detailed study. Rat females show hops, darts, and ear wiggles to initiate mating by males (66). These proceptive behaviors are considered an indicator of sexual desire and motivation. We found that female mice on a C57BL/6J background do not display these behaviors but will repeatedly approach the male to sniff or touch his head or genital region. While this behavior can elicit mounting behavior by the male, we found that approach behaviors by the female account for less than 20% of the variance in frequency of mounting by the male. Further, we cannot conclude that this approach behavior necessarily indicates sexual interest rather than social interest, since some of the females showing high numbers of approaches also rejected mounting attempts by hitting or biting the male. It is also possible that the overall reduction in ambulation by MC4RKO mice impacted their frequency of approaching the male (although see discussion of the behavior of tbMC4R^oxt^ mice below). Additional experimental designs used in rats to gauge female sexual motivation should be employed in future studies, such as social approach chambers comparing interest in intact and gonadectomized males, paced mating chambers, measurement of ultra-sonic vocalizations, conditioned place preference studies, or operant chambers to assess the number of nose pokes a female will exhibit to gain access to a male (67, 68).

The current study also revealed distinct roles of Sim1 and Oxt neurons in MC4R-dependent sexual behavior. Although expression of the MC4R solely on Sim1 neurons did not impact female investigation of the male, expression of the MC4R solely on oxytocin neurons significantly increased approaches by the female. Note that this effect occurred despite the reduced overall ambulatory activity of tbMC4R^oxt^ mice, which remained similar to that of MC4RKO mice. This finding suggests that locomotion and approach frequency are not necessarily coupled. Melanocortin action on PVH oxytocin neurons in particular could promote social interest, since these neurons are activated during social interaction in females (44, 69) and PVH oxytocin neuron numbers correlate with sociability in female mice (70). Indeed, the effect of an MC4R agonist that promoted social approach behavior in male and female mice was blocked by prior administration of an oxytocin receptor antagonist (71). The levels of social investigation in the MC4R^Oxt^ mice were higher than in animals with normal MC4R expression in all neurons suggesting that MC4R signaling may simultaneously suppress proceptive behaviors by acting on other neuron types. This hypothesis remains to be tested. Nevertheless, these results point to a complex but cumulatively positive influence of melanocortins on the motivation to engage in social and/or sexual interaction.

Our finding that MC4R signaling can influence rates of lordosis contradicts findings in rats. Previous findings involving administration of melanotan-II (MTII) or bremelanotide to rats implicate melanocortins in female solicitation behaviors but not lordosis (28, 72). However, estradiol and progesterone-primed rats exhibited a 100% lordosis quotient even without bremelanotide administration, so the potential for that drug to promote lordosis in a behavioral estrus state could not be evaluated. Administration of MTII faced a similar ceiling effect with a lordosis quotient of 100% +/- 0 with the drug and 93% +/- 3.0 with saline (72). In our study, we used ovariectomized mice treated with estradiol and progesterone to induce behavioral estrus; approximately 60% of mountings led to assumption of a lordotic posture in WT mice under these conditions. We did not examine mice given estradiol alone, a state of low sexual receptivity. In rats treated with estrogen only, MTII had no effect on the lordosis quotient or female solicitations (72), although bremelanotide did increase female solicitations (28). Species differences or different effects of endogenous versus exogenous melanocortins may underlie these divergent results.

We also found distinct roles of Sim1 and Oxt neurons in the MC4R-dependent promotion of lordosis. The lordosis quotient was normalized in mice that expressed MC4R exclusively on Sim1 neurons, strongly suggesting a role for Sim1 neurons in MC4R-dependent sexual behavior. In contrast, the tbMC4R^Oxt^ mice exhibited lordosis behavior that was intermediate between MC4RKO and control mice and was statistically indistinguishable from that of controls. These results suggest that the MC4R-dependent sexual behavior mediated by Sim1 neurons may be only partially dependent on oxytocin expressing neurons. Lordosis is facilitated dramatically by administration of oxytocin in rats, and oxytocin receptors in the mPOA and VMH appear to facilitate the frequency and duration of lordosis, respectively (73). However, oxytocin receptor knockout studies in mice suggest that oxytocin action may not be necessary for lordosis (74). This idea is consistent with our finding that Sim1 neuron MC4R re-expression had a more profound impact on female sexual behavior. Thus, multiple neuronal pathways may contribute to the lordotic response promoted by melanocortins.

### Obesity and sexual behavior

The MC4R is known to be involved in inducing satiety (75). Since MC4RKO mice exhibit obesity (47), we originally hypothesized that the metabolic effects of the absent MC4R might alter sexual behavior. However, weight-matched WT mice exhibited a lordosis quotient comparable to WT mice on a normal chow diet, implying that weight gain alone cannot explain the sexual deficit seen in MC4RKO mice. Indeed, tbMC4R^Sim1^ mice and tbMC4R^Oxt^ mice exhibited obesity that was as severe as that of MC4RKO mice, although those mouse lines had increased interest in the male and/or a normal lordosis quotient. This finding suggests that the abnormal metabolic function in MC4RKO mice cannot explain the impaired sexual behavior. It is likely that separate sexual and obesity-related melanocortin pathways exist.

Interestingly, female tbMC4R^Sim1^ mice did not have attenuated adiposity despite previous reports that ovary-intact, age-matched MC4R null females were heavier than tbMC4R^Sim1^ females (47). Chronic loss of estradiol production by the ovaries is known to increase body weight (76). Furthermore, estrogen receptor α has been shown to mediate this weight gain partially through regulation of POMC neurons, the source of melanocortins (77). The MC4RKO mice did not have a significant increase in fat mass in the month following ovariectomy (data not shown), which further implicates melanocortins in the anorexic effects of estrogens.

### The location of relevant melanocortin sensing neurons

Our findings raise the interesting question of where MC4R expressing Sim1 neurons that promote lordosis are located. Strong *Sim1* expression is found throughout the PVH, the supraoptic (SON) and posterior (PH) hypothalamic nuclei, and the NLOT. NLOT is a little studied area involved with chemosensory processing mediated by the main and the accessory olfactory systems. Lesions of this area in male rats interfere with copulation and other behaviors dependent on olfactory identification of other animals (78). Scattered expression of Sim1 is also found in the medial and basomedial amygdala, bed nucleus of the stria terminalis, medial preoptic nucleus, and in anterior and lateral hypothalamic areas, ventromedial thalamic and ventral and medial premammilary nucleus, ventral periaqueductal gray (PAG), and ventrolateral PAG accessory area (47). Additional work will be needed to determine which of these population(s) of melanocortin-responsive Sim1 neurons are involved in the female sexual response.

The partial similarity in the impact of deletion of MC4Rs from Sim1 and oxytocin neurons on lordosis could reflect gene deletion in a subset of oxytocin-positive Sim1 neurons, although non-overlapping populations with similar reproductive functions may exist. For example, MC4Rs are clearly expressed by Sim-1 neurons in the PVH (47–49). The question of whether PVH oxytocin neurons are responsive to melanocortins, however, has been an area of contention. Melanocortins induce immediate early gene expression in 14-15% of PVH oxytocin neurons in the mouse (38, 79). However, IHC and ISHH studies in mice found only a small PVH oxytocin neuronal population with MC4R-positive neurons (2.4% ± 0.4%) (80, 81), which accords with our findings. The discrepancy may reflect interaction between PVH MC4R neurons and PVH oxytocin neurons. For example, it has been suggested that MC4R-expressing glutamatergic neurons in the PVH may synapse on and activate oxytocin neurons (48). Additionally, release of oxytocin by a small number of melanocortin-responsive oxytocin neurons in the PVH may have a strong excitatory effect on the activity of surrounding oxytocin neurons (82, 83). Another area containing potential overlap of oxytocin and MC4R neurons is the SON. In rats, the SON receives projections from pro-opiomelanocortin (POMC) neurons (84–86) and contains high levels of the MC4R (87, 88). Accordingly, α-MSH induces Fos expression in rat oxytocin neurons in the SON and dendritic release of oxytocin, but it inhibits their firing rate and axonal secretion (89). In mice, the SON was originally reported to be devoid of the MC4R (80). Others, however, have reported MC4R expression in the SON of mice (90). MC4R expression in this area, however, appeared to be limited in our mice. Another area containing potential overlap of oxytocin and MC4R neurons is the medial amygdaloid nucleus, which contains many MC4R cells (80). The medial amygdala is important in sexual behavior particularly for the processing and integration of olfactory cues. Indeed, we also found colocalization between MC4R and Sim1 in the medial amygdala by IHC, an observation supported by previous findings (47, 48). Future studies should address which population(s) of melanocortin-responsive oxytocin neurons impact sexual behavior.

### Limitations

Our study has some important limitations. First, this study used mice with congenital deletion of the MC4R. It is possible that melanocortin circuits undergo rearrangement due to the lack of activation of the MC4R in some neurons, possibly altering the development of neural circuitry central to female sexual behavior. MC4R mRNA is first expressed at E12 in the neuroepithelium, the proliferative zone surrounding the lower portion of the third ventricle; thus, it is *theoretically* possible that the MC4R could be involved in hypothalamic neurogenesis (91). MC4R receptors are present and functional in the hypothalamus soon after birth (92), and could play a role in the development of neuronal projections in the hypothalamus, as has been shown for the Glp1R in Sim1 neurons (93). Another important consideration is that our findings are compatible with other neuropeptides and transmitters playing a key role in female sexual behavior independent of Sim1 circuitry. For example, kisspeptin neurons may play an important role in sexual motivation, including sexual preferences and lordosis (94). Kisspeptin neurons respond to melanocortins (95), suggesting they may function alongside or downstream of the circuits targeted in this study.

## Conclusions

Overall, we have demonstrated that MC4Rs are necessary for female mice to show normal interest in males and to exhibit a robust lordotic response to mounting. Furthermore, Sim1 neurons or oxytocin neurons are sufficient to maintain the lordotic response when MC4Rs are deleted from all other cells of the brain. The impact of MC4R on sexual behavior appears to be independent of its impact on body weight, offering hope that drug therapies to treat metabolic syndrome in women can avoid triggering alterations in their sexual experiences. Conversely, future elucidation of this neurocircuitry has the potential to provide more specific targets for female sexual dysfunction.

## Data availability statement

The raw data supporting the conclusions of this article will be made available by the authors, without undue reservation.

## Author contributions

Author ES was involved in the conception of the project, carrying out the experiments, and writing the manuscript. Authors MH, TC, and JB contributed to collecting data for the experiments. Author BX performed RNAscope experiments for MC4R^Sim1^ mice under the supervision of CL. Author AC was involved in the conception of the project, contributed to the experimental design, and assisted in carrying out experiments. MH and RR assisted with data analysis and visualization. Author JH supervised the entire project and she and MH edited the manuscript text. All authors contributed to the article and approved the submitted version.
